# The Satirist of the Stroke Spot: Cerebral Venous Sinus Thrombosis

**DOI:** 10.51894/001c.165589

**Published:** 2026-07-31

**Authors:** Caleb Eggenberger, Alan Lazzara

**Affiliations:** 1 Henry Ford - Jackson

## 77

### Introduction

Cerebral venous sinus thrombosis (CVST) is an uncommon pathology that often presents with a simple headache. However, patients can describe varied neurological symptoms attributable to the location and size of the occlusion(s). We discuss an elderly female initially thought to be suffering an ischemic stroke or seizure and discovered to have extensive CVST.

### Case Description

A 79 year-old female presents via EMS with left upper extremity weakness and dysarthria. Patient also describes convulsions where her head, left arm, and left leg shook. This was witnessed by her husband and she denies any loss of consciousness during the event. Two days prior, she experienced a severe sudden onset posterior headache. Her PCP diagnosed her with a sinus infection – despite the lack of sinus drainage, pressure, or fever and was given diclofenac and promethazine for symptom relief. Later that evening, she developed transient left eye lateral field diplopia which she attributed to the promethazine.

Emergent stroke evaluation ensues. CT head reads negative for mass or intracranial hemorrhage; discussion around thrombolytics occurs with the patient and husband. CTA head and neck shows a superior sagittal CVST extending into the right transverse sinus and right internal jugular vein. Heparin infusion and levetiracetam bolus dose are given and the patient is admitted to step down. She transitions to apixaban and after an uneventful 3 days is discharged with hematology follow-up.

### Discussion

Patient’s initial presentation was concerning for hemorrhagic versus ischemic stroke, and thus she was activated on the rapid stroke pathway for possible initiation of thrombolytic therapy. While getting ready to administer the thrombolytics, radiology called with a stat update to report identification of CVST after the initial CT was read as normal. Had this critical finding not been relayed, the patient would have received unnecessary thrombolytic therapy, which would have exposed her to additional risks such as intracranial hemorrhage. CVST can easily mimic other types of neurologic pathology. Our case highlights the importance of obtaining a thorough history and exam, angiography in a stroke work up, and timely communication with a radiologist to ensure the appropriate diagnosis is reached.

